# Ultrafine Particles and Mortality: A Scoping Review of Epidemiologic Evidence and Exposure Assessment Limitations

**DOI:** 10.3390/toxics14080664

**Published:** 2026-07-27

**Authors:** Humza Rashid, Edward Wilson, Haya Alhmly, Amy A. Hunter, Wig Zamore, Misha Eliasziw, Doug Brugge

**Affiliations:** 1 Department of Public Health Sciences, University of Connecticut School of Medicine, Farmington, CT 06030, USA; hrashid@uchc.edu (H.R.); amhunter@uchc.edu (A.A.H.); 2 General Surgery Residency Program, University of Connecticut School of Medicine, Farmington, CT 06030, USA; edwilson@uchc.edu; 3 Jean Mayer USDA Human Nutrition Research Center on Aging, Tufts University, Boston, MA 02111, USA; haya.alhmly@tufts.edu; 4 Somerville Transportation Equity Partnership, Somerville, MA 02145, USA; wigzamore@gmail.com; 5 Department of Public Health and Community Medicine, Tufts University, Boston, MA 02111, USA

**Keywords:** ultrafine particles, mortality, air pollution, particle number concentration, exposure misclassification, scoping review

## Abstract

Ultrafine particles (UFPs; <100 nm) can penetrate biological barriers and trigger inflammation, oxidative stress, and endothelial dysfunction, yet they remain largely unregulated and understudied relative to PM_2.5_. This scoping review, conducted following PRISMA-ScR guidelines, systematically searched PubMed and Scopus for epidemiological studies examining ambient UFP exposure and mortality. Of 704 articles screened, 21 studies published between 2007 and 2025 met inclusion criteria. Fourteen assessed short-term exposure (≤1 month) using time-series or case-crossover designs with central-site particle number concentration monitors, and seven assessed long-term exposure using land-use regression, chemical transport, or machine learning models. A majority of both short-term (10 of 14) and long-term (6 of 7) studies reported positive associations between UFP exposure and mortality, particularly for respiratory outcomes. The strongest associations were observed in studies using higher-resolution or source-specific exposure methods, a pattern consistent with reduced misclassification. However, exposure assessment approaches varied widely in spatial resolution, instrument type, and particle size definitions, introducing substantial heterogeneity that limits comparability across studies. These findings underscore the need for standardized UFP measurement protocols, high-resolution exposure assessment, and further investigation of disparities in exposure and health outcomes to inform regulatory consideration.

## 1. Introduction

Ultrafine particles (UFPs), defined as particulate matter with an aerodynamic diameter of less than 100 nanometers, are increasingly recognized as a hazardous component of ambient air pollution. Their small size and large surface area per unit mass allow them to penetrate deep into the lungs, translocate into the bloodstream, and cross the blood–brain barrier. They interact with multiple organ systems [1]. Toxicology studies have shown that UFPs can induce oxidative stress, systemic inflammation, and endothelial dysfunction: all biologically plausible mechanisms for increased risk of mortality [2].

Although the health effects of particulate matter, particularly PM_2.5_ and PM_10_, are well-established and have shaped global air quality regulations [3,4], UFPs remain largely unregulated and under-characterized in terms of their independent health impacts. This is, in part, because of the challenges of assigning exposure due to high temporal and spatial variation in UFP concentrations. Nevertheless, a growing number of epidemiological studies have examined associations between UFP exposure and mortality. However, heterogeneity in exposure assessment, outcome definitions, methods for estimating associations, and population characteristics limit comparability across studies and reduce confidence in the accuracy of reported associations.

Two recent systematic reviews by Bergmann and colleagues have advanced the evidence base on UFP exposure and health. The first examined short-term UFP exposure and mortality using random-effects meta-analysis and found no statistically significant pooled associations with natural, cardiovascular, or respiratory mortality [5]. The second provided a comprehensive review of long-term UFP exposure and a broad range of health outcomes, including mortality, cardiovascular and respiratory morbidity, inflammatory markers, and neurological endpoints [6]. Although this long-term review reported a pooled hazard ratio of 1.06 (95% CI: 1.04–1.08) for natural mortality, this estimate was based on only three studies with high between-study heterogeneity (I^2^ = 77.4%), and the authors rated confidence in the evidence as “low.” Cardiovascular and respiratory mortality could not be meta-analyzed because fewer than four effect estimates were available. Both reviews acknowledged substantial heterogeneity in UFP measurement methods across studies.

A central challenge for any quantitative synthesis of UFP health evidence is that the studies being pooled measure UFPs in fundamentally different ways. Across the literature, particle size ranges vary from as narrow as 10–100 nm to as broad as 3–800 nm, depending on the instrument used. Condensation particle counters, scanning mobility particle sizers, and differential mobility particle sizers differ in their lower and upper size cutoffs, and inter-instrument comparison studies have documented systematic biases of 20–80% for the same aerosol [7,8]. Because particle number concentration is dominated by the smallest nucleation-mode particles, even small differences in the lower size cutoff produce large differences in the reported concentration.

Standardizing effect estimates to a common increment addresses unit scaling but does not resolve the fact that what constitutes a “particle” differs across studies. Bergmann et al. [6] acknowledged this directly, stating that different surrogates of UFPs measured as PNC “were not converted to make them more comparable and hence used interchangeably.”

To our knowledge, no scoping review has specifically examined UFP exposure and mortality. Scoping reviews serve a different purpose than systematic reviews: rather than producing a pooled summary, they map the nature, features, and extent of the available evidence, identify patterns across studies, and highlight gaps in the literature [9]. Given the measurement heterogeneity described above, we believe a scoping review is the appropriate framework for this evidence base at this stage of the science.

We conducted this scoping review, following PRISMA-ScR guidelines, which includes both short-term and long-term UFP exposure studies and mortality outcomes. Our review places particular emphasis on exposure assessment strategies and how differences in measurement approaches may shape the patterns observed across studies. We present an interpretation of the evidence that differs from the recent systematic reviews in two respects: first, we find that a majority of short-term studies report positive associations with mortality, a finding that is obscured when studies are pooled at a single lag; and second, we argue that the pattern of stronger associations in studies using higher-resolution exposure methods warrants careful attention as a hypothesis for future research, rather than being treated as an artifact of heterogeneity.

## 2. Methods

### 2.1. Review Design and Framework

Our scoping review was conducted in accordance with the Preferred Reporting Items for Systematic Reviews and Meta-Analyses extension for Scoping Reviews (PRISMA-ScR) guidelines. Consistent with the purpose of a scoping review, our objective was to map the range and nature of the available evidence, categorize studies by key methodological features, and identify patterns and gaps in the literature, rather than to produce pooled summary estimates or to formally evaluate individual study quality [9]. The primary objective was to characterize the current literature examining the association between ambient UFP exposure and mortality outcomes in human populations. This scoping review had a particular focus on exposure assessment strategies and estimation of associations, while also including study design and mortality endpoint issues. Definitions and abbreviations for the technical terms and measurement metrics used throughout this review are provided in Table 1.

### 2.2. Eligibility Criteria

Studies were included if they were original, peer-reviewed epidemiological investigations that evaluated the association between ambient UFP exposure and mortality. Eligible studies involved human populations, including an unambiguous assessment of UFPs as an exposure of interest, and reported mortality outcomes, including all-cause or cause-specific mortality.

Studies were excluded if they focused solely on morbidity, hospitalization, or subclinical health outcomes without addressing mortality. Additional exclusion criteria included studies that measured only larger particulate fractions (e.g., PM_2.5_ or PM_1_) without specifically assessing UFPs. Studies were also excluded if they used vague or inconsistent definitions of ultrafine particles, such as “nanoparticles”, without alignment to the under-100-nanometer standard. Studies reporting particle number concentration (PNC) without an explicit size range were retained, as PNC is widely recognized as a valid surrogate for ultrafine particles. Studies based on animal models or in vitro experiments, or non-original research formats, were also excluded.

### 2.3. Information Sources and Search Strategy

A comprehensive literature search was conducted in two electronic databases: PubMed and Scopus. The search strategy combined text word terms related to ultrafine particles (“ultrafine particles”, “particle matter”, “PM.1”, “particle number count”) and mortality (“mortality”, “death”, “lifespan”, “mortality rate”, “death rate”) using Boolean operators to optimize search sensitivity. The search was limited to articles published in English. The final search of all databases was conducted on 31 July 2025. The complete search strategy for both databases is presented in Table 2.

### 2.4. Study Selection

The screening process was conducted in two stages by two independent reviewers (H.R. and H.A.). First, titles and abstracts were reviewed to exclude irrelevant studies. Second, the full texts of potentially eligible articles were reviewed in detail to confirm inclusion based on the predefined eligibility criteria. Disagreements were resolved through discussion and consensus.

### 2.5. Data Extraction and Synthesis

Data from included studies were extracted independently by two authors (H.R. and H.A.). Extracted variables included study location, design, population characteristics, method of UFP exposure assessment, type of mortality outcome (e.g., all-cause, cardiovascular, respiratory), reported estimates of association, and covariates used in the analysis. Disagreements were resolved through discussion and consensus. For the purposes of this review, short-term exposure was defined as an exposure window of one month or less (e.g., daily values, lag structures), while long-term exposure referred to any duration exceeding one month, including annual averages and multi-year cumulative exposures. Given the heterogeneity across studies regarding exposure assessment strategies, mortality definitions, and analytical approaches in estimating associations, meta-analysis was not deemed to be feasible. Consistent with standard scoping review methodology, we did not conduct formal quality assessment or risk-of-bias evaluation of individual studies, as the goal was to map the breadth and characteristics of the evidence rather than to weight findings by methodological rigor [9,10]. Instead, we performed a narrative synthesis that categorized studies by design, exposure assessment method, geographic region, and outcome, and described patterns across these groupings. This synthesis also identifies methodological challenges and gaps in the literature to guide future epidemiologic research and regulatory evaluation.

During the preparation of this manuscript, the authors used ChatGPT 5.5 (OpenAI, San Francisco, CA, USA) to assist with language editing and to help draft and refine wording of portions of the text during revision. The tools were not used for the literature search, study screening, data extraction, or interpretation of findings; all scientific content, analyses, and conclusions were developed, verified, and approved by the authors, who take full responsibility for the work.

## 3. Results

### 3.1. Study Selection

A total of 704 articles were identified through database searches of PubMed (n = 387) and Scopus (n = 317). After removing 190 duplicates, 514 unique articles remained for title and abstract screening. Of these, 462 were excluded for not meeting inclusion criteria, such as lacking UFP as an exposure or mortality as an outcome. The remaining 52 articles underwent full-text review to assess eligibility based on predefined criteria.

**Figure 1:**
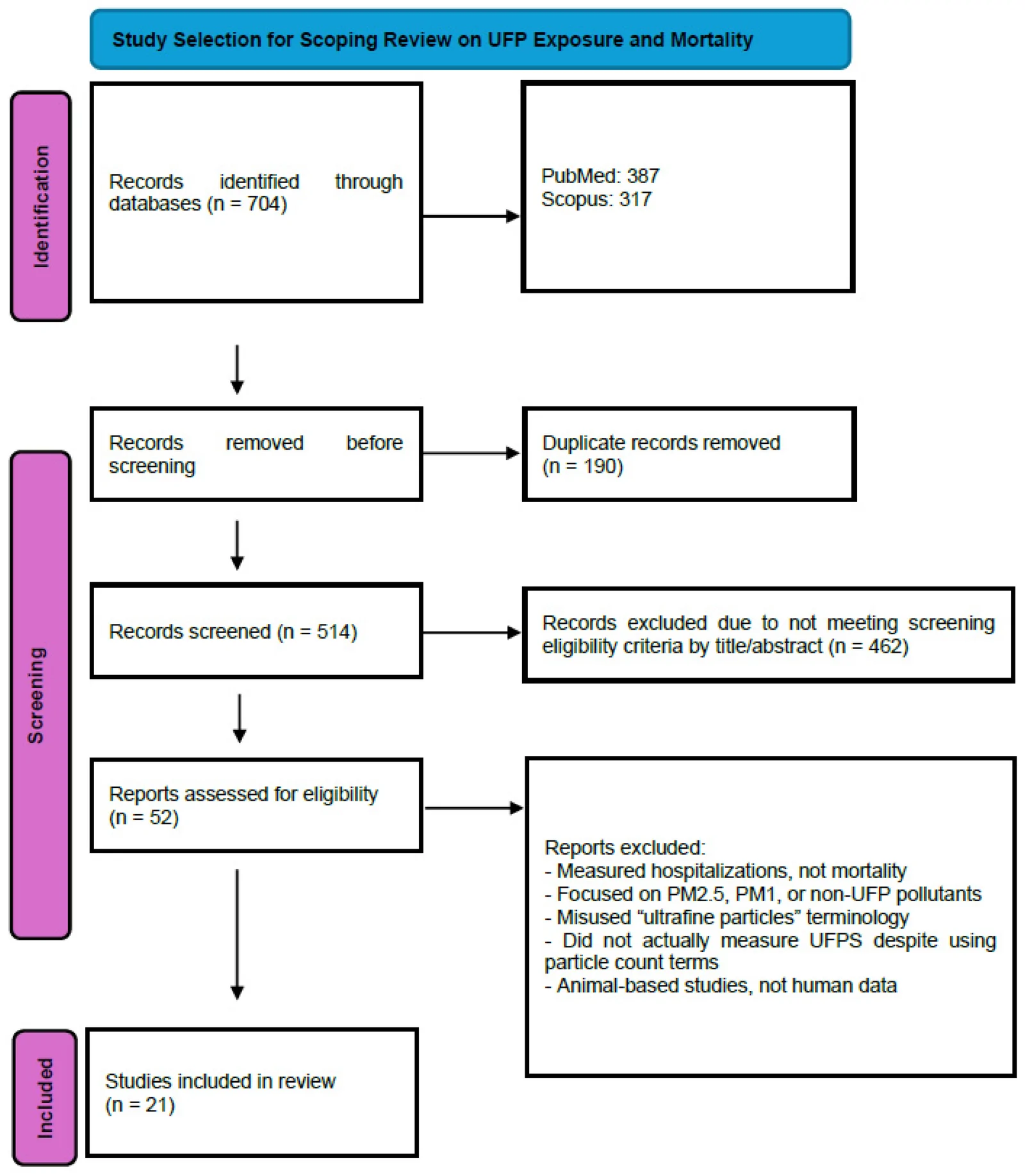
PRISMA 2020 Flow Diagram.

Thirty-one studies were excluded at the full-text stage for one or more of the following reasons: (1) the study assessed only morbidity, hospitalization, or subclinical outcomes; (2) it focused on pollutants such as PM_2.5_ or PM_1_; (3) the study used only vague terms such as “nanoparticles” without alignment to under-100-nanometer standard or (4) the study was conducted in animal or in vitro models. The remaining 21 studies met all eligibility criteria and were retained in the final synthesis. The complete selection process is illustrated in the PRISMA 2020 flow diagram (Figure 1).

### 3.2. Study Characteristics

**Figure 2:**
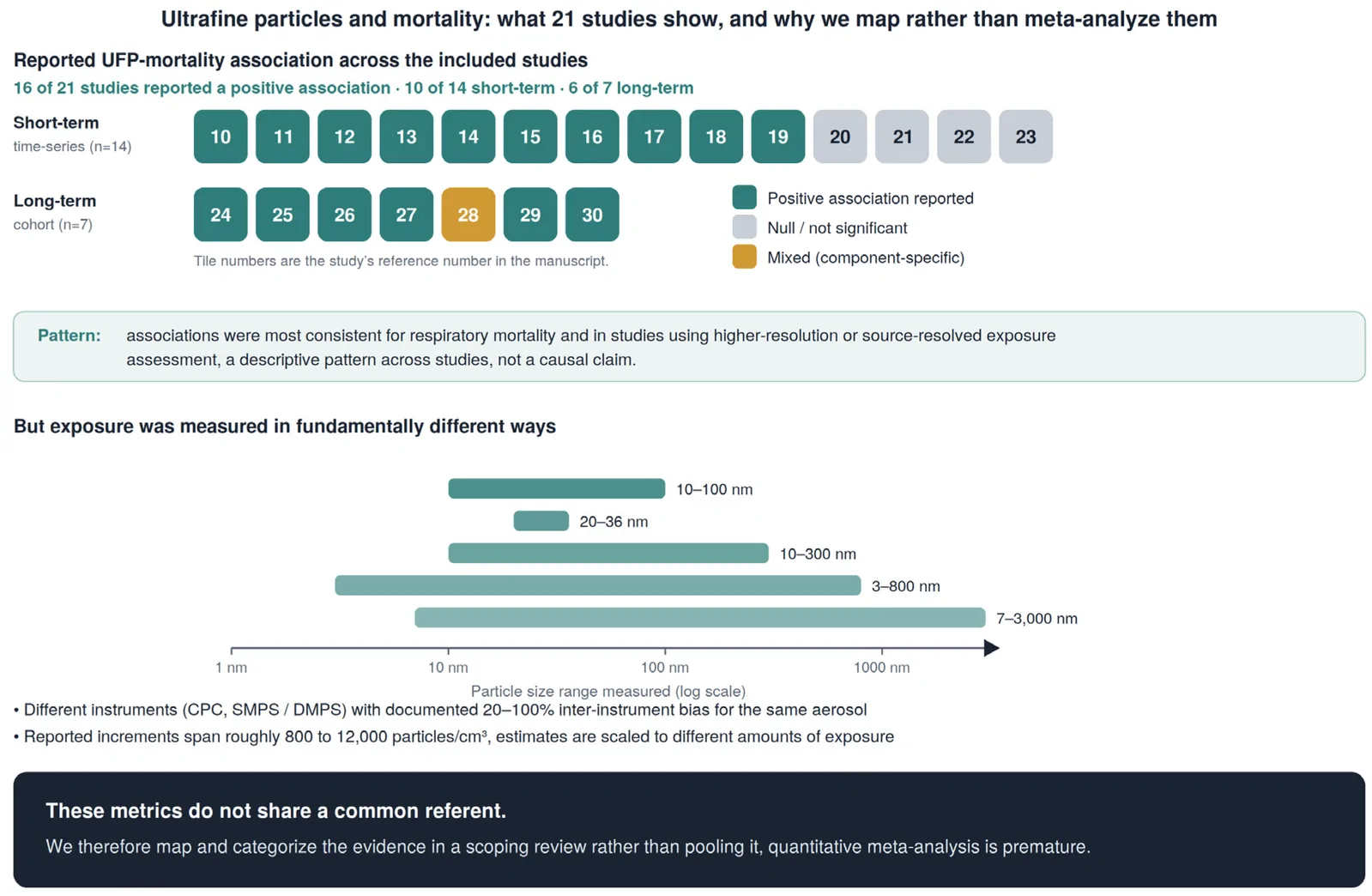
Most included studies reported positive UFP-mortality associations, but the studies measured UFP over different particle-size ranges with different instruments and increments, which makes quantitative pooling premature and motivates a scoping-review approach.

Included studies were published between 2007 and 2025. Of the 21 studies included (Table 2 and Table 3), 14 assessed short-term exposure [11,12,13,14,15,16,17,18,19,20,21,22,23,24] and 7 assessed long-term exposure to UFPs in relation to mortality [25,26,27,28,29,30,31]. The studies were conducted across a range of geographic locations, including North America [25,26,27,28,29,30,31], Europe [11,12,13,14,15,16,17,18,19,20,21], and Asia [24], and varied considerably in study design, sample size, follow-up duration, exposure assessment, and estimation of association. Figure 2 maps the 21 included studies by design and by direction of the reported association.

Short-term studies were predominantly time-series analyses [12,13,14,15,16,17,18,19,20,21] or case-crossover designs [11] that investigated daily fluctuations in particle number concentrations (PNC) and their associations with daily mortality counts (Table 3). All 21 studies included all-cause mortality, with 12 also reporting cause-specific outcomes, including mortality related to cardiovascular [11,13,17,18,20,25,26,27,28,29,30,31] and respiratory causes [17,18,20,25,26,27,28,30]. Stroke [26,28] and cancer [25,30] mortality were examined in only a small number of studies.

### 3.3. Exposure Assessment Strategies

**Figure 3:**
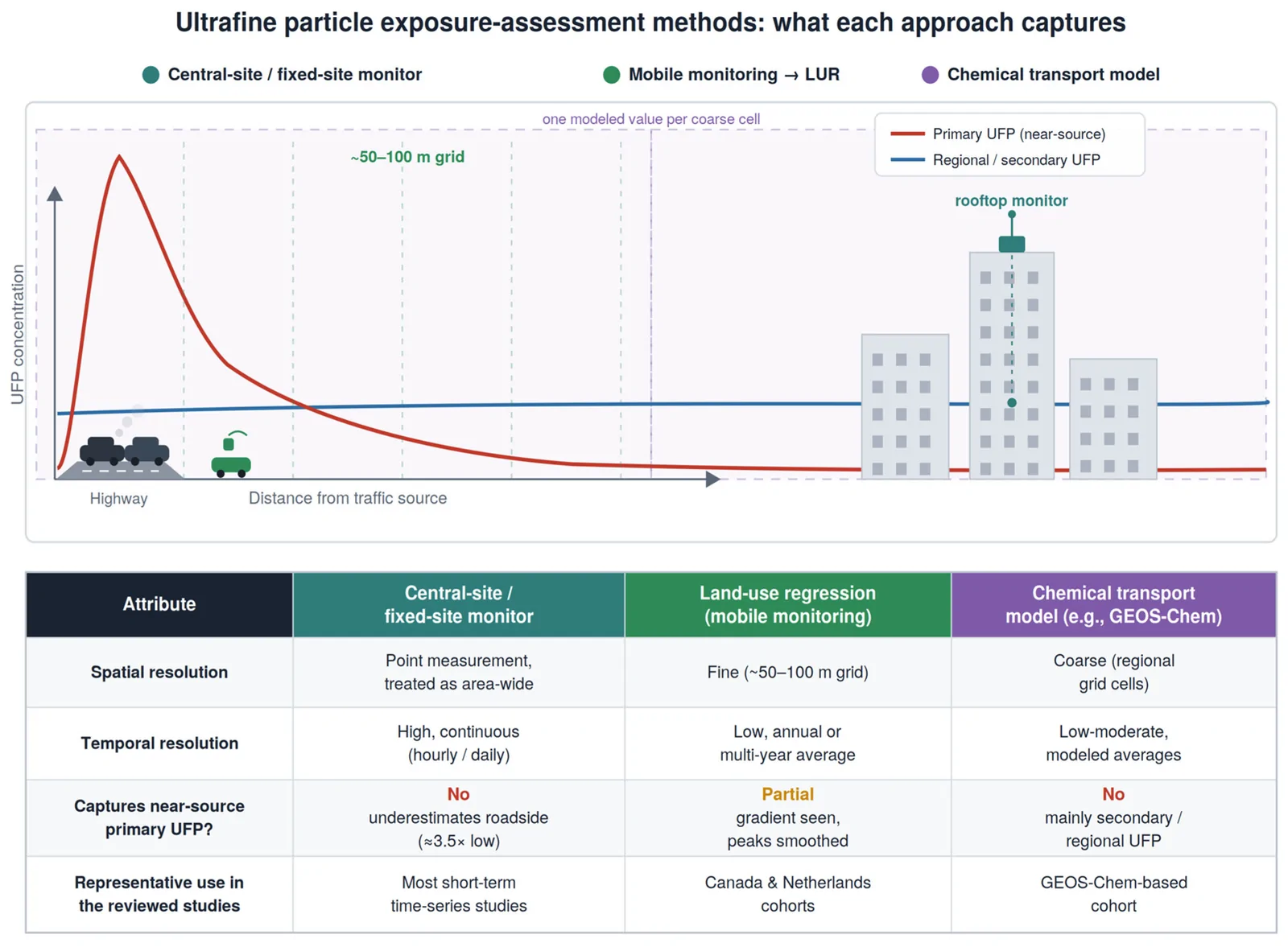
UFP exposure-assessment methods differ in spatial and temporal resolution and in whether they capture near-source primary UFP, a key source of the measurement heterogeneity that limits direct comparison of estimates across studies.

The 21 studies included in this scoping review employed a range of strategies to estimate exposure to UFPs, leading to substantial heterogeneity in spatial resolution, source attribution, and measurement accuracy (Table 4). Many studies used particle number concentration (PNC) as a proxy for UFPs [11,12,13,14,15,16,17,18,19,20,21], which is widely accepted as the vast majority of particles captured by PNC are <100 nm. However, the instruments, lower limit size cut-offs, and monitoring strategies varied widely, likely affecting the denominator in exposure assignment. Figure 3 compares these approaches schematically.

In short-term studies, exposure was measured at fixed-site monitors in all short-term studies, often located on rooftops or in background urban locations [11,12,13,14,15,16,17,18,19,20,21,22,23,24]. While these monitors provided continuous temporal data that reflected trends in UFP concentrations over large geographic areas, they were unable to capture spatial variability that would estimate absolute concentrations of near-source UFP (e.g., from traffic). For instance, rooftop monitors in Copenhagen reported UFP levels that were estimated to be 3.5× lower than street-level concentrations [11]. A related consideration is that the studies did not use a consistent monitoring-station type: some relied on urban-background monitors intended to represent area-wide concentrations, whereas others used traffic-influenced or roadside monitors [17,21], and at least one multi-site study mixed background and traffic-adjacent stations across cities [13]. Because near-source concentrations are not representative of area-wide population exposure, combining background and near-source monitors across studies introduces a further source of non-comparability; for cross-study comparison, monitoring stations should ideally be of a single, consistent type, either representative of a broader urban area or of a defined near-source environment, rather than a mixture of the two.

Exposure assessment among the long-term studies varied in spatial resolution and methodological rigor. Studies using mobile monitoring data and high-resolution land-use regression (e.g., 100 m grid models) provided more granular exposure assignments [25,27,28,30]. These methods were, however, still limited in terms of resolving near-highway gradients, did not assess indoor concentrations, were aggregated into annual averages, and were not adjusted for time-activity locations. Coarser models, such as GEOS-Chem-based chemical transport simulations, were able to capture only regional variation [26]. Models of this sort also could not resolve near-road gradients, thus likely measuring secondary UFP while missing peak exposures of primary UFP.

Only a few studies attempted to characterize source-specific or traffic-adjacent exposure, such as including traffic-related monitoring sites [17] and using positive matrix factorization (PMF) to differentiate UFPs originating from fresh traffic emissions [14,18].

The use of high-resolution LUR models based on mobile monitoring campaigns, as in the studies from Canada and the Netherlands [25,27,28,30], allowed for improved differentiation of exposure gradients across neighborhoods. However, even these models had limited resolution of roadside gradients, failing to capture transient peak exposures, such as during rush hour, as well as other hyperlocal sources of combustion. This is partly because these mobile monitoring designs prioritized broad spatial coverage rather than repeatedly sampling specific locations with intense emissions. As a result, exposure estimates were often averaged over grid cells of 100 m for an annual average. This may smooth out the steep ultrafine particle gradients that occur within 10 s of meters of busy roadways and change within hours [6]. At the same time, resolving near-road concentration peaks does not by itself yield a better estimate of population exposure. Because individuals spend only a small fraction of the day in close proximity to busy roadways, exposure is more accurately represented by a time-weighted average across the microenvironments a person occupies throughout the day, including home, workplace, commute, and indoor settings, than by peak concentrations measured at a single high emission location. Exposure metrics that emphasize roadside maxima may therefore misrepresent typical personal exposure even when their spatial resolution is high, a limitation that applies to fixed roadside monitors and high-resolution models alike.

In contrast, studies that incorporated traffic-specific monitors used roadside stations or positive matrix factorization (PMF) to capture more localized source-specific exposures and reported more pronounced contrasts in UFP concentrations across space and time [14,17].

More broadly, the resolution of spatial models varied substantially across studies. For example, Qi et al. [26] relied on GEOS-Chem chemical transport models with a relatively coarse spatial grid of 27 by 27 km, which was unable to capture urban-scale and neighborhood-level differences in exposure. In contrast, Bouma et al. [25], Lloyd et al. [28], and Weichenthal et al. [27] employed high-resolution exposure models with 100 m grid cells derived from mobile monitoring and machine learning algorithms, enabling finer detection of urban-scale variability.

While the majority of the studies in the scoping review attempted to quantify UFP exposure at population or neighborhood levels, no study incorporated personal monitoring data or validated modeled exposures with individual-level measurements. Moreover, few studies accounted for time-activity patterns [31], indoor infiltration [31], indoor emission sources such as cooking [32], or the effect of ventilation systems [32], which can substantially influence personal exposure levels.

### 3.4. Analytical Approaches in Estimating Associations

A variety of analytical approaches were used to estimate associations between exposure and mortality. The most common approach among the short-term studies was Poisson regression, with three studies using generalized additive models [11,14,19] and six accounting for overdispersion [12,13,14,18,20,21]. Despite a common Poisson regression approach, the resulting relative risk estimates are not comparable, as the reported increase in relative risk per change in UFP concentration ranged from 2750 particles/cm^3^ to 12,033 particles/cm^3^. Cox proportional hazards regression was used in almost all the long-term studies, but the reported increase in hazard ratio per change in UFP concentration ranged from 834 particles/cm^3^ to 10,000 particles/cm^3^, again resulting in estimates of association that are not comparable.

### 3.5. Short-Term Exposure and Mortality

Ten of the fourteen short-term studies reported positive associations between UFP and mortality [11,12,13,14,15,16,17,18,19,20]. Six of these studies identified lagged effects [11,12,13,18,19,20], while the remaining studies reported mixed or null findings [14,15,16,17,18,19]. In addition to exposure misclassification, variability in study design and analytical approaches contributed to heterogeneity across the short-term studies. Although most studies used time-series, with one case-crossover study [11], their different analytical approaches influenced both the estimation of associations and how lag structures were modeled, particularly when adjusting for temperature, seasonality, or co-pollutants such as PM_2.5_, NO_2_, or black carbon. Finally, the range of changes in UFP concentrations associated with estimates of effects complicates direct comparison of the magnitudes of association.

### 3.6. Long-Term Exposure and Mortality

The seven long-term studies used large population-based cohorts. These studies typically assessed annual average UFP exposure over multiple years [25,26,27,28,29,30], with the exception of Ostro et al. [10], which used monthly modeled estimates, with follow-up periods ranging from six to fifteen years and populations ranging from 100,000 to over 10 million individuals. Six of seven long-term studies reported positive associations between UFP exposure and mortality [25,26,27,28,29,30]. Only one study reported mixed findings, with significant associations limited to specific ultrafine particle components rather than total concentrations [10]. Among these, associations with respiratory mortality were reported more frequently than associations with cardiovascular mortality. Studies that used higher-resolution spatial models (e.g., 50–100 m grid LUR) [25,27,28,30] tended to report larger effect estimates than those using coarser models, though differences in study populations, geographic settings, and co-pollutant adjustment strategies may also contribute to this pattern. Importantly, emerging evidence suggests there may be disparities in long-term health impacts of UFPs by race, income, and geography [26]. This is an area that deserves more research and underscores the need for equity-focused exposure science.

### 3.7. At-Risk Populations and Disparities

Several studies highlighted the uneven distribution of health risks from UFP exposure, with evidence suggesting that certain demographic and geographic subpopulations showed larger reported UFP-mortality associations [26,27,28]. These disparities were often driven by proximity to emission sources such as major roadways, industrial zones, and dense urban corridors. Areas such as these typically have lower property values due to noise, pollution, and other undesirable features, and are therefore more likely to be inhabited by individuals of lower socioeconomic status, with higher percentages of racial and ethnic minorities and immigrants.

In the long-term study by Qi et al. [26], race and ethnicity emerged as moderators of UFP-associated mortality risk. The investigators found that non-Hispanic Black individuals had substantially higher reported UFP-associated non-accidental mortality than non-Hispanic White individuals, while Hispanic populations also showed elevated associations. These disparities persisted even after adjusting for socioeconomic status and comorbidities, underscoring the potential for environmental justice implications of air pollution exposure.

Other studies found that the association between UFP exposure and mortality was stronger in lower-income neighborhoods, even after adjusting for co-pollutants and baseline health characteristics [27,28], as well as larger reported associations among individuals with lower income, lower educational attainment, or immigrant status [13,16,18,29]. These findings suggest that socioeconomic disadvantage and residential location may be associated with larger reported UFP–mortality associations, highlighting the need for equity-focused air pollution research and interventions. Together, these findings suggest that these patterns may coincide with, and could compound, existing health inequities, reflecting both physiological susceptibility and broader patterns of social and environmental disadvantage.

## 4. Discussion

### 4.1. Summary of Main Findings

This scoping review mapped and categorized 21 epidemiological studies examining associations between ambient UFP exposure and mortality. Across both exposure windows, a majority of studies reported positive associations between UFP exposure and all-cause mortality, with the most frequently reported cause-specific associations involving respiratory mortality. Studies varied considerably in exposure assessment methods, analytical approaches, and the magnitude and precision of reported associations.

Differences in the resolution of spatial exposure models may contribute to the observed variation in reported associations. While non-differential exposure misclassification typically attenuates associations toward the null under classical measurement error assumptions, the error structure in air pollution epidemiology is often more complex. Berkson-type error, which is common when modeled area-level exposures are assigned to individuals, does not bias point estimates but reduces statistical power. Differential misclassification, in which exposure error is correlated with outcome determinants, can bias associations in either direction.

This directional ambiguity is particularly relevant for UFPs. The conventional assumption in air-pollution epidemiology is that non-differential exposure misclassification biases associations toward the null; however, for near-roadway ultrafine particles specifically, prior work has shown that misclassification can operate in the opposite direction, so that the true association may be either under- or overstated depending on how exposure is assigned [33].

Cancer mortality may be underrepresented in the current literature because detecting associations requires longer follow-up periods and larger sample sizes. The limited examination of stroke mortality is less clearly explained by study design constraints, as stroke can be triggered by short-term exposure, and may instead reflect the field’s predominant focus on broader cardiovascular and respiratory endpoints [1,2].

More broadly, the included studies varied by more than two orders of magnitude in population and event counts from cohorts of roughly 100,000 to over 10 million individuals, and from a few thousand to nearly one million deaths; this variation in statistical power is one reason why associations of similar magnitude reached statistical significance in some studies but not others.

### 4.2. This Review in Relation to Recent Systematic Reviews

To our knowledge, this is the first scoping review specifically examining UFP exposure and mortality. Two recent systematic reviews by Bergmann and colleagues [5,6] have addressed related questions using different methods, and it is important to clarify both the relationship and the distinction between their work and ours.

As noted above, systematic reviews with meta-analysis and scoping reviews are complementary rather than competing approaches, and the choice between them depends on whether the underlying evidence base is sufficiently standardized to support quantitative pooling.

Our scoping review takes a different approach. By categorizing studies according to their exposure assessment methods and describing the pattern of findings within each category, rather than pooling across categories, we find that positive associations with mortality are reported in a majority of both short-term (10 of 14) and long-term (6 of 7) studies. We also observe that studies using higher-resolution or source-specific exposure methods tend to report larger associations. We present this as a descriptive pattern warranting further investigation, not as a causal finding.

In sum, the Bergmann reviews provide valuable comprehensive catalogs of the UFP health literature and apply important structured assessment tools. Our review contributes a different lens: one that foregrounds measurement heterogeneity as a defining feature of the evidence base, argues that this heterogeneity is a barrier to quantitative pooling rather than a limitation to be noted and set aside, and maps the literature in a way that can inform the design of future studies with more comparable exposure metrics.

### 4.3. Research Gaps and Future Directions

This scoping review highlights several critical gaps in the current literature that should help guide future research on UFP exposure and mortality. First, there is a need for standardized definitions and measurement protocols. Studies varied widely in the particle size ranges they classified as ultrafine, in the instruments and models they used to assess them, in how they processed the exposure data, and in the analytical approaches used to estimate the associations. Without harmonization, it will remain difficult to compare effect estimates of association across studies and to conduct pooled analyses that can inform policy.

In addition, exposure assessment must evolve beyond the use of single-site monitors or models with coarse grids. Future studies should incorporate mobile monitoring campaigns, personal exposure devices for a subset of study participants, and high-resolution spatial and temporal models that account for residential location, time-activity patterns, and infiltration into indoor spaces. These methods could reduce exposure misclassification and more accurately capture the exposure of individuals living near traffic corridors or industrial zones. Although real-time personal monitoring is not feasible in large population-based studies, it may be possible to validate exposure assignment by collecting personal monitoring data from a subsample and comparing those values with model-based estimates [31]. This type of hybrid validation could improve confidence in exposure models.

As fresh traffic emissions have been associated with larger mortality estimates than secondary aerosols or photochemically aged particles, techniques such as positive matrix factorization and source-resolved modeling should be used more widely to clarify which sources contribute most to health risks.

Lastly, more research is needed to characterize disparities in exposure and outcomes. While a few studies examined differential effects by race, income, or immigration status, most did not. Understanding who is most affected by UFP exposure is essential for designing equitable interventions and informing environmental justice efforts.

### 4.4. Strengths and Limitations of This Review

This scoping review provided a structured synthesis of the current epidemiological literature examining UFP exposure and mortality. Focusing specifically on mortality outcomes and including both short- and long-term studies, our scoping review offered a targeted perspective on mortality, the most serious health endpoint associated with air pollution. The inclusion of studies across multiple countries and diverse urban environments improved generalizability and highlighted both global consistencies and regional differences.

A key strength of our scoping review is the detailed attention to exposure assessment methods. By categorizing how studies defined, measured, and modeled UFPs, and by describing how these methodological features relate to the pattern of reported associations, this review adds interpretive details that are not present in the recent systematic reviews by Bergmann and colleagues [5,6]. Our explicit argument that measurement heterogeneity precludes reliable meta-analysis represents an alternative scientific perspective that we believe contributes to the discourse on how best to evaluate UFP health evidence.

At the same time, this scoping review has limitations. It was restricted to peer-reviewed, English-language publications indexed in PubMed and Scopus. Relevant studies published in other languages or in gray literature may have been missed. Consistent with scoping review methodology and PRISMA-ScR guidelines, we did not conduct formal quality assessment or risk-of-bias evaluation of individual studies [9]. Readers should consider the quality of individual studies when interpreting the patterns described here.

Because the search was limited to English-language articles indexed in PubMed and Scopus, the evidence summarized here is subject to language and database selection and to potential publication bias, as studies reporting null or negative associations are less likely to be published or indexed. These constraints bear on generalizability: the patterns described here can be generalized to the English-language, indexed literature on which they are based, and may not represent evidence from settings, such as many low- and middle-income regions, that are under-represented in these databases.

The observation that higher-resolution exposure studies tend to report stronger associations is a descriptive pattern, not a causal conclusion, and should be treated as a hypothesis for future testing.

## 5. Conclusions

This scoping review found that a majority of epidemiological studies reported positive associations between UFP exposure and mortality, with the most frequently reported associations involving respiratory outcomes. Our categorization of the evidence by exposure assessment method suggests that the pattern of findings may vary with measurement quality, though this observation requires confirmation in future studies designed to test it directly.

Our interpretation differs from that of recent systematic reviews and meta-analyses, which found null pooled associations for short-term exposure and fragile, low-confidence associations for long-term exposure. We attribute this difference to the limitations of pooling studies that use fundamentally different UFP measurement methods.

Despite established biological plausibility and growing epidemiological evidence, ultrafine particles remain unregulated by ambient air quality standards. Additional research should prioritize standardized measurement, high-resolution exposure assessment, source apportionment, and the systematic assessment of disparities in exposure and health outcomes.

## Figures and Tables

**Table 1:** Definitions and abbreviations of key terms and measurement metrics used in this review.

Term/Abbreviation	Definition	Size Range/Unit
Ultrafine particles (UFP)	Airborne particles < 100 nm in aerodynamic diameter; can deposit deep in the lung and translocate systemically.	<100 nm
Particle number concentration (PNC)	Count of airborne particles per unit volume of air; used as a surrogate for UFP because most counted particles are <100 nm.	particles/cm^3^
PM2.5/PM10	Particulate matter ≤ 2.5 µm/≤10 µm in aerodynamic diameter, defined and regulated by mass.	µg/m^3^
UFP mass/species	UFP exposure expressed as mass rather than number, sometimes resolved by chemical species (e.g., elemental or organic carbon, copper).	µg/m^3^
Reported UFP size ranges	Lower and upper size cut-offs vary by instrument and study.	10–100, 20–36, 10–300, up to 3–800 and 7–3000 nm
Condensation particle counter (CPC)	Counts particles after growing them in a supersaturated vapor; a common PNC monitor.	particles/cm^3^
Scanning/differential mobility particle sizer (SMPS/DMPS)	Classifies and counts particles by electrical mobility to produce size-resolved number distributions.	particles/cm^3^
Central-site/fixed-site monitor	Stationary monitor (often rooftop or urban background) giving continuous concentrations over a broad area but limited spatial detail.	Depends on monitor
Land-use regression (LUR)	Statistical model predicting pollutant concentrations across space from land-use, traffic, and monitoring inputs.	Depends on model
Chemical transport model (CTM)	Simulation (e.g., GEOS-Chem) estimating concentrations from emissions and atmospheric processes, typically at coarse spatial resolution.	Depends on model
Mobile monitoring	Repeated measurements collected from vehicles to map fine-scale spatial variation in concentrations.	Depends on model
Positive matrix factorization (PMF)	Source-apportionment method resolving measured particles into contributing sources (e.g., fresh traffic vs. secondary aerosol).	Depends on model
Time-series/case-crossover	Short-term designs relating day-to-day variation in exposure to daily mortality counts.	Depends on study
Cohort study/Cox proportional hazards	Longitudinal design following individuals over time; hazard ratios from Cox regression relate long-term exposure to mortality.	Depends on study
Exposure misclassification	Error in assigning exposure to individuals; non-differential error usually biases associations toward the null, though near-source UFP error can act in either direction.	Depends on model
Interquartile range (IQR)	Difference between the 75th and 25th percentiles of the exposure distribution; effect estimates are often scaled per IQR increase.	Depends on model

**Table 2:** Search Strategy for PubMed and Scopus.

Database	Search Strategy	Results
PubMed	(“ultrafine particles”[tw] OR “particle matter”[tw] OR PM.1 OR “particle number count”[tw]) AND (lifespan OR mortality OR death OR “mortality rate”[tw] OR “death rate”[tw])	387
Scopus	TITLE-ABS-KEY(“ultrafine particles” OR “particle matter” OR PM.1 OR “particle number count”) AND TITLE-ABS-KEY(lifespan OR mortality OR death OR “mortality rate” OR “death rate”)	317
Total		704

Search conducted on 31 July 2025. No formal date restrictions were applied. Results were limited to English-language publications. [tw] = text word field tag in PubMed.

**Table 3:** Overview of Study Design, Population, and Analytical Methods.

**(a) Short-Term Studies**					
**Citations**	**Region/City**	**Data Source**	**Study Design**	**Statistical Analysis**	**Population and Size**
Bergmann et al., 2023 [11]	Denmark	The National Health Registers, 2002–2018	Time-stratified case-crossover study	Main effect estimates: Conditional logistic regression (case-crossover). Sensitivity/exposure–response exploration → generalized additive model with Poisson distribution.	Adults aged ≥30 in Greater Copenhagen; 140 k non-accidental deaths
Breitner et al., 2009 [12]	Erfurt, Germany	Death certificate records from local health authorities, 1995–2004	Time-series study	Quasi-Poisson regression models	Residents of Erfurt; 17,713 non-infant, non-accidental deaths
Stafoggia et al., 2017 [13]	Finland, Sweden, Denmark, Germany, Italy, Spain, and Greece	Eight European centers, 1999–2013	Multi-center time-series study	Time-series analysis using quasi-Poisson regression, followed by random-effects meta-analysis	Residents of eight European cities; ~3–80 nonaccidental daily deaths per city; city-specific daily mortality counts over ≥3 years
Tobías et al., 2018 [14]	Barcelona, Huelva, and Santa Cruz de Tenerife, Spain	Urban background central monitoring stations, Barcelona, Huelva, Santa Cruz de Tenerife (2009–2014)	Time-series study	Generalized additive models with quasi-Poisson regression	Entire populations of three cities; daily counts of non-accidental mortality. Approx. total deaths: Barcelona (~93 k), Huelva (~14 k), Tenerife (~16 k) during 2009–2014 (~123 k deaths combined).
Olstrup et al., 2019 [15]	Stockholm, Sweden	Swedish Central Bureau of Statistics, 2000–2016	Time-series study	Poisson regression models	Stockholm residents; ~0.8–0.9 million; 6210 days of data
Samoli et al., 2016 [16]	London, UK	National death registrations, 2011–2012	Time-series study	Poisson regression models	Residents of London; daily counts of non-accidental, cardiovascular, and respiratory deaths (~84 k total deaths; ~25 k cardiovascular; ~12 k respiratory across 2011–2012).
Schwarz et al., 2023 [17]	Dresden, Leipzig, and Augsburg, Germany	The official statistics from three German cities, 2010–2017	Multi-city time-series study	Poisson regression and multilevel meta-analysis	Residents of Dresden, Leipzig, and Augsburg; 100 K+ total deaths: 84 k natural, 44 k cardiovascular, 6 k respiratory
Rivas et al., 2021 [18]	Barcelona, Helsinki, London, Zurich	The official health statistics services for the 4 cities, 2000–2010	Multi-city time-series study	Generalized linear models with quasi-Poisson regression	Urban populations in each city; daily mortality counts from 2010 to 2016 (varies slightly by site); cause-specific mortality included cardiovascular and respiratory deaths. Median daily natural deaths ranged from 19 (Helsinki) to 123 (London); CVD deaths from 7 (Helsinki/Zurich) to 37 (London); respiratory deaths from 1 (Helsinki/Zurich) to 17 (London).
Stölzel et al., 2007 [19]	Erfurt, Germany	Death certificates, local health authorities	Time-series study in Erfurt, Germany (1995–2001)	Poisson Generalized Additive Models	~200,000 residents; 10,762 total deaths, 8561 cardio-respiratory deaths
Lanzinger et al., 2016 [20]	Dresden and Augsburg, Prague, Ljubljana and Chernivtsi	Official statistics and monitoring data, Augsburg 2011–2012; Dresden 2011–2012; Prague 2012–2013; Ljubljana 2012–2013; Chernivtsi 2013–2014	Multi-center time-series study	Time-series analysis across five Central European cities (from the UFIREG project), using city-specific quasi-Poisson regression models and random-effects meta-analysis	Daily mortality from five cities (Augsburg, Dresden, Prague, Ljubljana, Chernivtsi); ~40 k total nonaccidental deaths across study periods (city totals ≈ 5.2 k, 8.7 k, 19.6 k, 4.2 k, 2.3 k)
Wolf et al., 2015 [21]	Augsburg, Germany	MONICA/KORA myocardial infarction registry, 2013–2018	Time-series study	Quasi-Poisson regression models	Approximately 400,000 individuals aged 25–74 years residing in Augsburg, Germany; daily counts of natural, cardiovascular, and respiratory deaths
Kettunen et al., 2007 [22]	Helsinki, Finland	Statistics Finland, 1998–2004	Time-series study	Poisson regression models	Residents aged ≥65 in the Helsinki metropolitan area; 3265 stroke deaths (1304 in warm season, 1961 in cold season)
Peters et al., 2009 [23]	Erfurt, Germany	Death certificate records from local health authorities, 1991–2002	Time-series study	Poisson regression with lags 0–5; time-varying coefficient models to assess changing mortality risks per IQR pollutant increase.	~200,000 residents; daily mortality counts from city death registries
Breitner et al., 2021 [24]	Beijing, China	The Beijing Center for Disease Control (CDC)	Time-series study (May–December 2008; before, during, after Olympics).	Quasi-Poisson regression	Urban Beijing residents; daily counts of respiratory mortality (~1900 deaths total, including ~500 pneumonia deaths) across May–December 2008.
**(b) Long-Term Studies**					
**Citations**	**Region/City**	**Data Source**	**Study Design**	**Statistical Analysis**	**Population and Size**
Bouma et al., 2023 [25]	Netherlands	Statistics Netherlands, 2013–2019	Nationwide cohort study	Cox proportional hazards models	10.8 million adults aged ≥30; 945,615 deaths over 71 million person-years
Qi et al., 2024 [26]	New York State, USA	Upstate NY and NYC Vital Records, 2013–2019	Long-term cohort study	A modified difference-in-difference model	1010 county subdivisions; 883,725 non-accidental deaths
Weichenthal et al., 2022 [27]	Montreal and Toronto, Canada	CanCHEC cohort; adults ≥ 25 years, mortality follow-up 2001–2016	Nationwide cohort study	Cox proportional hazards models	2.45 million adults in the Canadian Census Health and Environment Cohort (CanCHEC); 300 k+ deaths over follow-up
Lloyd et al., 2024 [28]	Montreal and Toronto, Canada	The Canadian CensusHealth and Environment Cohort (CanCHECs)	Nationwide cohort study	Cox proportional hazards models	1.54 million adults aged 25–90 living in Toronto or Montreal; 174,240 non-accidental deaths over 22.8 million person-years
Ostro et al., 2015 [10]	California, USA	California Teachers Cohort, 2001–2007	Prospective cohort study	Cox proportional hazards models	>100,000 women (mean age 57), primarily non-Hispanic white
Pond et al., 2023 [29]	USA	The U.S. National Health Interview Survey (NHIS), 1986–2015	Nationwide cohort study	Cox proportional hazards models	617,966 U.S. adults aged 18–84 from NHIS with mortality follow-up via National Death Index; 103,454 deaths including 25,694 from cancer and 41,967 cardiopulmonary deaths
Vachon et al., 2025 [30]	Quebec City, Canada	Quebec Integrated Chronic Disease Surveillance System (QICDSS), 2000–2017	Retrospective open population-based cohort	Cox proportional hazards	715,157 adults aged ≥20 residing in Quebec City from 2000 to 2017; 74,511 all-cause deaths including 15,603 from ischemic heart disease over 7.2 million person-years

Note: Population and size data reflect what was reported in the original studies. Some studies reported total population, others reported total deaths, and some reported both.

**Table 4:** Summary of Exposure Metrics, Methods of Assessment, Limitations, and Outcomes.

**(a) Short-Term Studies**							
**Citations**	**UFP Metric and Particle Size Range**	**Exposure Assignment Method**	**Exposure Assessment**	**All-Cause Mortality**	**Specific-Cause Mortality**	**Captures Near-Source Primary UFP?**	**Reported Increment (Unit)**
Bergman et al., 2023 [11]	Daily PNC (11–700 nm); 30 min intervals averaged to daily means	Fixed-site rooftop urban background monitor (~20 m elevation) in central Copenhagen; 15 km radius coverage; DMPS (Vienna-type DMA + TSI CPC 3010); street-level UFP ~3.5× higher	Single monitor for entire area; no spatial modeling; no adjustment for time-activity or residential mobility	OR 1.05 (95% CI 1.00–1.11)	COPD: OR 1.22 (95% CI 1.05–1.41); Cardiovascular: HR 1.08 (95% CI 0.97–1.21)	No—monitor location and design did not capture traffic-adjacent exposure	IQR: 3075 Particles/cm^3^
Breitner et al., 2009 [12]	Daily mean PNC (10–100 nm); daily averages used for short- and cumulative-lag models	Fixed-site urban background station located ~1 km south of city center, ~40 m from major road; aerosol spectrometer with Vienna-type DMA + TSI CPC 3022	Monitor not street-level; no spatial modeling or adjustment for time-activity	RR 1.06 (95% CI 1.01–1.11) per IQR 7649 particles/cm^3^ (15-day cumulative mean)	Not Reported	No—although located near a road, the monitor was not street-level and did not resolve individual-level or hyperlocal exposure patterns	IQR: 7649 Particles/cm^3^
Stafoggia et al., 2017 [13]	Daily PNC as proxy for UFPs; size ranges varied by site (10–100 nm in Helsinki, 7–3000 nm in Rome); 24 h averages from hourly/sub-hourly data	Fixed-site monitors in 8 European cities (urban/suburban background; Rome traffic-influenced); instruments included CPCs and SMPS/DMPS	No spatial modeling or personal exposure correction; no adjustment for time-activity or residential mobility	%IR 0.35 (95% CI −0.05, 0.75) per 10,000 particles/cm^3^ (lag 6)	Not reported (no stable quantitative estimate)	Mixed—only Rome captured traffic-adjacent UFP exposure; other sites likely underrepresented near-road emissions	per 10,000 Particles/cm^3^
Tobías et al., 2018 [14]	Daily average PNC segregated into N1 (vehicle exhaust) and N2 (secondary UFPs) using black carbon-based segregation method	Fixed-site urban background monitors in Barcelona, Huelva, and Tenerife; CPCs (TSI 3785 in Barcelona; TSI 3776 in Huelva and Tenerife)	Monitors not traffic-adjacent; no spatial modeling; no adjustment for time-activity or residential mobility	Barcelona: 1.63% (95% CI 0.74, 2.52) per IQR 3277 cm^−3^ (lag 0); Huelva: 3.95% (95% CI 0.10, 7.95) per IQR 12,033 cm^−3^ (lag 0)	Not Reported	Partially—N1 approximates traffic-related UFPs, but monitoring sites were not traffic-adjacent and exposure not assigned at individual level	IQR: Various Particles/cm^3^
Olstrup et al., 2019 [15]	PNC (10–200 nm); PNC_4_ (<4 nm) modeled via regression with PNC_7_; ~70% data coverage; 5 min intervals averaged to hourly then daily means	Fixed-site rooftop monitor in central Stockholm (~20 m elevation); TSI CPC 3022A	No spatial modeling or individual-level assignment; no adjustment for residential mobility or time-activity (e.g., commuting, window-opening)	Not Significant	Not Reported	No—single central monitor may misclassify exposure due to poor capture of near-road UFP variability	IQR: 5354 Particles/cm^3^
Samoli et al., 2016 [16]	Daily UFP number size distribution from four source-specific factors (traffic, urban background, nucleation, secondary) identified using Positive Matrix Factorization; ~14 size bins <0.6 nm; 24 h averages from continuous monitoring	Fixed-site central urban background monitor (North Kensington, London); SMPS (TSI 308 classifier + TSI 3075 CPC); total PNC from CPC (TSI 3022)	Single-site exposure; no spatial modeling, time-activity adjustment, or residential mobility correction	Not significant (NSD sources)	Not reported (no significant NSD estimates)	No—monitoring was performed at a background site without resolution of traffic-adjacent exposure	IQR: 5180 Particles/cm^3^
Schwarz et al., 2023 [17]	UFPs (10–100 nm) and PNCs (10–800 nm); daily mean concentrations calculated from hourly data	Six fixed-site monitors in three German cities (4 urban background, 2 traffic-related) as part of the German Ultrafine Aerosol Network (GUAN); mobility particle size spectrometers	No spatial modeling, individual-level exposure assignment, or adjustment for time-activity/residential mobility	Not significant	Respiratory: 4.46% (95% CI 1.52, 7.48) per IQR 3223 particles/cm^3^ (lag 5–7); Cardiovascular: not significant	Partially—included both urban background and traffic-related monitors; peak exposures better captured than single-monitor studies	IQR: 3223 Particles/cm^3^
Rivas et al., 2021 [18]	Daily PNC by source type (fresh traffic, urban background, secondary aerosols, photonucleation) identified using Positive Matrix Factorization; UFPs 10–100 nm; 5 min intervals aggregated to daily means	Fixed-site rooftop urban background monitor in Barcelona (~20 m elevation); TSI CPC 3022A	No spatial modeling, personal exposure assignment, or adjustment for time-activity/residential mobility	Helsinki: 1.3% (95% CI 0.07, 2.5) per IQR PNC (lag 2); Barcelona, London, Zurich: not significant	Cardiovascular: Barcelona 3.7% (95% CI 0.17, 7.4) lag 1; Zurich 3.8% (95% CI 0.31, 7.4) lag 0; others not significant. Respiratory: London 2.6% (95% CI 0.84, 4.45) lag 3; Zurich 9.4% (95% CI 1.0, 17.9) lag 1; Helsinki 4.7% (95% CI 0.11, 9.5) lag 0; Barcelona: reduced risk (−8.6%, 95% CI −14.5, −2.4) lag 2.	No—monitors were not traffic-adjacent and did not resolve near-road exposure gradients	IQR: Various Particles/cm^3^
Stölzel et al., 2007 [19]	Daily mean PNC (10–100 nm); ~10% data imputed	Fixed-site urban background monitor in Erfurt, ~1 km from city center, ~40 m from nearest major road; aerosol spectrometer combining DMPS and LAS-x	No spatial modeling or adjustment for time-activity	RR 1.029 (95% CI 1.003–1.055) per IQR 9748 particles/cm^3^ (lag 4)	Cardio-respiratory: RR 1.031 (95% CI 1.003–1.060) per IQR 9748 particles/cm^3^ (lag 4); Respiratory: not significant	No—urban background location likely underrepresents near-roadway exposure	IQR: 9748 Particles/cm^3^
Lanzinger et al., 2016 [20]	Daily mean PNC (10–300 nm); measured using condensation particle counters; 5 min resolution aggregated to 24 h means	Fixed-site urban background stations in 5 Central European cities (UFIREG project); CPC 3022a at all sites	No spatial modeling or individual-level exposure assignment; no adjustment for time-activity or residential mobility	Percent change: −0.3% (95% CI −3.8, 3.2) per IQR 2750 particles/cm^3^ (lag 0–5)	Cardiovascular: −0.2% (95% CI −5.5, 5.4); Respiratory: 9.9% (95% CI −6.3, 28.8) per IQR 2750 particles/cm^3^ (lag 0–5)	No—monitor placement and lack of spatial modeling did not capture traffic-adjacent exposures	IQR: 2750 Particles/cm^3^
Wolf et al., 2015 [21]	Hourly PNC (10–800 nm); 5 min intervals aggregated to daily means; source apportionment by wind direction	Fixed roadside monitor near major intersection in Augsburg; TSI CPC 3022A	No spatial modeling or personal exposure linkage; no adjustment for time-activity or residential mobility	Not reported	Recurrent MI: 6.0% (95% CI 0.6, 11.7) per IQR 5529 particles/cm^3^ (5-day average)	Partially—monitor was traffic-adjacent and captured near-road UFP peaks, but exposures were not assigned at the individual or address level	IQR: 5529 Particles/cm^3^
Kettunen et al., 2007 [22]	24 h median ultrafine PNC (<100 nm); monitor location changed during study period	Two fixed-site rooftop monitors in Helsinki (~20 m above ground, ~100–300 m from major roads); Differential Mobility Particle Sizer (DMPS), University of Helsinki	No spatial modeling, individual-level exposure assignment, or adjustment for time-activity/residential mobility	Not reported	Stroke: 8.5% (95% CI −1.2, 19.1) per IQR UFP (lag 1, warm season)	No—exposure captured at background or elevated locations with limited near-road resolution	IQR: 7330 Particles/cm^3^
Peters et al., 2009 [23]	Daily PNC (10–100 nm); data available 1995–2002; 10 min resolution aggregated to daily means	Two fixed-site government/GSF urban background rooftop monitors in Erfurt (~20 m elevation, ~2 km apart); University of Helsinki DMPS	No spatial modeling, personal exposure assignment, or adjustment for time-activity/residential mobility	2.9% (95% CI 0.3, 5.5) per IQR 9743 particles/cm^3^ (lag 4)	Not Reported	No—monitor was not traffic-adjacent and likely underrepresented near-source primary UFP peaks	IQR: 9743 Particles/cm^3^
Breitner et al., 2021 [24]	Daily PNC (10–100 nm) and size-segregated UFPs (3–800 nm); hourly data aggregated to daily averages; IQR of total PNC: 7958 particles/cm^3^	Fixed-site rooftop urban background station on Peking University campus; Twin Differential Mobility Particle Sizer (DMPS; 3–800 nm)	No spatial modeling or adjustment for time-activity	Not reported	Respiratory: 16.3% (95% CI 4.3, 26.5) per IQR 7958 particles/cm^3^ (lag 7); Pneumonia: elevated, strongest during Olympics (not fully quantified)	No—monitor was not traffic-adjacent and likely underrepresented near-source primary UFP peaks	IQR: 7649 Particles/cm^3^
**(b) Long-Term Studies**							
**Citations**	**UFP Metric and Particle Size Range**	**Exposure Assignment Method**	**Exposure Assessment**	**All-Cause Mortality**	**Specific-Cause Mortality**	**Captures Near-Source Primary UFP?**	**Reported Increment (Unit)**
Bouma et al., 2023 [25]	Annual average UFP (10 nm range, particles/cm^3^) at baseline residential address; 1-year temporal resolution	National land-use regression (LUR) model based on 2016–2017 mobile monitoring campaigns (14,392 road segments + regional background sites); exposures assigned to residential addresses	No direct monitoring at participant homes; no personal exposure data; no adjustment for time-activity	HR 1.012 (95% CI 1.010–1.015) per IQR 2723 particles/cm^3^	Cardiovascular: HR 1.005 (95% CI 1.000–1.011); Respiratory: HR 1.022 (95% CI 1.013–1.032); Lung cancer: HR 1.038 (95% CI 1.028–1.048) per IQR 2723 particles/cm^3^	Partially—spatial resolution is high, but mobile campaign did not include roadside hotspots or personal exposure	IQR: 2723 Particles/cm^3^
Qi et al., 2024 [26]	Annual average UFP concentrations (10–100 nm) modeled at ~17-mile resolution; hourly output aggregated to annual averages by county subdivision; exposure categorized into interquartile range and high-level (≥75th percentile)	GEOS-Chem-APM chemical transport model at ~17 × 27 km resolution (0.25° × 0.3125°)	Coarse spatial resolution (~17 × 27 km); exposure not validated by direct monitoring at individual level; no adjustment for time-activity or residential mobility	RR 1.10 (95% CI 1.05–1.17) per high UFP exposure (≥75th percentile)	Cardiovascular: RR 1.11 (95% CI 1.05–1.18); Cerebrovascular: RR 1.21 (95% CI 1.10–1.35); Pulmonary heart disease: RR 1.33 (95% CI 1.13–1.57); Respiratory: RR 1.09 (95% CI 1.00–1.18); Mental disorders, Nervous system: not significant	No—coarse model grid did not resolve near-road UFP gradients or traffic-adjacent exposures	IQR: 834 Particles/cm^3^
Weichenthal et al., 2024 [27]	Annual average UFP (20–36 nm); exposures assigned to residential postal codes and averaged over follow-up	National land-use regression (LUR) model at 100 m resolution from 10 Canadian cities (2016–2017 mobile monitoring); Montreal LUR from 2011 to 2012 mobile monitoring; back-extrapolated to 2001–2015	No direct monitoring during study period (1999–2015); no adjustment for time-activity or residential mobility	HR 1.07 (95% CI 1.02–1.12) per 10,000 particles/cm^3^	Cardiovascular: HR 1.05 (95% CI 1.01–1.10); Respiratory: HR 1.04 (95% CI 1.00–1.08); others not significant	Partially—spatial resolution was high, but mobile campaigns lacked direct measurements of traffic-adjacent microenvironments	per 10,000 Particles/cm^3^
Lloyd et al., 2024 [28]	Annual average UFP number concentration (particles/cm^3^) at city-block (~100 m) resolution	Hybrid land-use regression and machine learning models from 2020 to 2021 mobile monitoring, back-cast to 1998–2016; UFPs (20–36 nm) modeled from 2011 to 2012 mobile monitoring; assigned to residential postal codes in Toronto and Montreal	No direct residential monitoring; no adjustment for time-activity or mobility	HR 1.073 (95% CI 1.061–1.085) per 10,000 particles/cm^3^	Respiratory: HR 1.174 (95% CI 1.130–1.220); ischemic heart disease: HR 1.094 (95% CI 1.062–1.126); Cerebrovascular: not significant; Lung cancer: not significant	Yes—high-resolution spatial modeling and on-road monitoring captured traffic-related exposure patterns effectively	per 10,000 Particles/cm^3^
Ostro et al., 2015 [10]	Monthly modeled UFP mass and species (e.g., elemental carbon, organic carbon, copper); hourly model output averaged to monthly values	UCD/CIT Source-Oriented Chemical Transport Model at 4 km resolution; adjusted for residential history	No direct monitoring or instruments used; no adjustment for time-activity patterns	Not significant	Cardiovascular: UF Cu HR 1.03 (95% CI 1.00–1.05); UF high-sulfur fuel HR 1.04 (95% CI 1.01–1.07). IHD: UF mass HR 1.10 (95% CI 1.02–1.18); UF SOA_ant HR 1.25 (95% CI 1.13–1.39); UF EC HR 1.15 (95% CI 1.06–1.26); UF other metals HR 1.13 (95% CI 1.05–1.21). Pulmonary: not significant	Partially—source-oriented chemical transport model resolved UFP by emission source and species, but 4 km grid resolution limits capture of street-level gradients	IQR: Various Particles/cm^3^
Pond et al., 2022 [29]	Annual PNC estimates for 2017; modeled using empirical land-use regression (LUR) trained on mobile and fixed-site monitoring (n = 118 sites); explained ~77% of spatial variability (CV R^2^ = 0.72)	National census-block resolution; exposures assigned by census tract using population-weighted block averages	Assumed to proxy lifetime exposure; no adjustment for time-activity or residential mobility; noted temporal misalignment between 2017 PNC and 1986–2014 survey data	HR 1.03 (95% CI 1.02–1.04) per IQR 5007 particles/cm^3^	Cardiopulmonary: HR 1.04 (95% CI 1.02–1.06); Cancer: HR 1.05 (95% CI 1.02–1.08) per IQR 5007 particles/cm^3^	Partially—mobile monitoring and LUR captured spatial gradients at national scale, but exposure estimates were annual averages, not near-road specific, and lacked individual-level precision	IQR: 5007 Particles/cm^3^
Vachon et al., 2025 [30]	Annual mean UFP concentrations at 50 × 50 m resolution; prediction surfaces generated using statistical (GAM, FSLR) and machine learning (XGBoost, Random Forest) methods; model performance up to CV R^2^ = 0.86	2019–2020 mobile monitoring data; exposures assigned at 6-digit postal code level	No adjustment for time-activity or long-term residential mobility	HR 1.04 (95% CI 1.02–1.05) per IQR UFP (XGBoost, single-pollutant); HR 1.09 (95% CI 1.06–1.11) per IQR (multi-pollutant)	IHD: HR 1.02 (95% CI 0.98–1.05), not significant (single-pollutant); HR 1.09 (95% CI 1.04–1.15) (multi-pollutant)	Yes—mobile monitoring captured near-road face UFP variation; high-resolution modeling incorporated traffic intensity, proximity to roads, and industrial activity; estimates reflect primary traffic-related exposures at final spatial scales	IQR: Various Particles/cm^3^

## Data Availability

No new data were created or analyzed in this study. Data sharing is not applicable to this article.

## References

[1] Ohlwein S., Kappeler R., Kutlar Joss M., Künzli N., Hoffmann B. (2019). Health effects of ultrafine particles: A systematic literature review update of epidemiological evidence. Int. J. Public Health.

[2] Kuntic M., Kuntic I., Cleppien D., Pozzer A., Nußbaum D., Oelze M., Junglas T., Strohm L., Ubbens H., Daub S. (2025). Differential inflammation, oxidative stress and cardiovascular damage markers of nano- and micro-particle exposure in mice: Implications for human disease burden. Redox Biol..

[3] Atkinson R., Mills I.C., Walton H.A., Anderson H.R. (2015). Fine particle components and health—A systematic review and meta-analysis of epidemiological time series studies of daily mortality and hospital admissions. J. Expo. Sci. Environ. Epidemiol..

[4] Nemmar A., Hoet P.H.M., Vanquickenborne B., Dinsdale D., Thomeer M., Hoylaerts M.F., Vanbilloen H., Mortelmans L., Nemery B. (2002). Passage of inhaled particles into the blood circulation in humans. Circulation.

[5] Bergmann M.L., Haddad-Thoelke P., Jeong H., Kappeler R., Altug H., Boogaard H., Joss M.K., Lim Y.-H., Loft S., Andersen Z.J. (2025). Systematic review and meta-analysis on short-term concentrations of ambient ultrafine particles and natural, cardiovascular and respiratory mortality. Environ. Res..

[6] Bergmann M., Haddad-Thoelke P., Jeong H., Kappeler R., Altug H., Oberwinster L., Boogaard H., Pohl T., Soppa V., Ogurtsova K. (2026). Systematic review and meta-analysis on the health effects of long-term exposure to ultrafine particles. Eur. Respir. Rev..

[7] Asbach C., Kaminski H., Fissan H., Monz C., Dahmann D., Mülhopt S., Paur H.R., Kiesling H.J., Herrmann F., Voetz M. (2009). Comparison of four mobility particle sizers with different time resolution for stationary exposure measurements. J. Nanopart. Res..

[8] Zimmerman N., Godri Pollitt K.J., Jeong C.H., Wang J.M., Jung T., Cooper J.M., Wallace J.S., Evans G.J. (2014). Comparison of three nanoparticle sizing instruments: The influence of particle morphology. Atmos. Environ..

[9] Tricco A.C., Lillie E., Zarin W., O’Brien K.K., Colquhoun H., Levac D., Moher D., Peters M.D.J., Horsley T., Weeks L. (2018). PRISMA Extension for Scoping Reviews (PRISMA-ScR): Checklist and explanation. Ann. Intern. Med..

[10] Ostro B., Hu J., Goldberg D., Reynolds P., Hertz A., Bernstein L., Kleeman M.J. (2015). Associations of mortality with long-term exposures to fine and ultrafine particles, species and sources: Results from the California Teachers Study cohort. Environ. Health Perspect..

[11] Bergmann M.L., Andersen Z.J., Massling A., Kindler P.A., Loft S., Amini H., Cole-Hunter T., Guo Y., Maric M., Nordstrøm C. (2023). Short-term exposure to ultrafine particles and mortality and hospital admissions due to respiratory and cardiovascular diseases in Copenhagen, Denmark. Environ. Pollut..

[12] Breitner S., Stölzel M., Cyrys J., Pitz M., Wölke G., Kreyling W.G., Küchenhoff H., Heinrich J., Wichmann H.-E., Peters A. (2009). Short-term mortality rates during a decade of improved air quality in Erfurt, Germany. Environ. Health Perspect..

[13] Stafoggia M., Schneider A., Cyrys J., Samoli E., Andersen Z.J., Bedada G.B., Bellander T., Cattani G., Eleftheriadis K., Faustini A. (2017). Association between short-term exposure to ultrafine particles and mortality in eight European urban areas. Epidemiology.

[14] Tobías A., Rivas I., Reche C., Alastuey A., Rodríguez S., Fernández-Camacho R., de la Campa A.M.S., de la Rosa J., Sunyer J., Querol X. (2018). Short-term effects of ultrafine particles on daily mortality by primary vehicle exhaust versus secondary origin in three Spanish cities. Environ. Int..

[15] Olstrup H., Johansson C., Forsberg B., Åström C. (2019). Association between mortality and short-term exposure to particles, ozone and nitrogen dioxide in Stockholm, Sweden. Int. J. Environ. Res. Public Health.

[16] Samoli E., Atkinson R.W., Analitis A., Fuller G.W., Beddows D., Green D.C., Mudway I.S., Harrison R.M., Anderson H.R., Kelly F.J. (2016). Differential health effects of short-term exposure to source-specific particles in London, U.K. Environ. Int..

[17] Schwarz M., Schneider A., Cyrys J., Bastian S., Breitner S., Peters A. (2023). Impact of ambient ultrafine particles on cause-specific mortality in three German cities. Am. J. Respir. Crit. Care Med..

[18] Rivas I., Vicens L., Basagaña X., Tobías A., Katsouyanni K., Walton H., Hüglin C., Alastuey A., Kulmala M., Harrison R.M. (2021). Associations between sources of particle number and mortality in four European cities. Environ. Int..

[19] Stölzel M., Breitner S., Cyrys J., Pitz M., Wölke G., Kreyling W.G., Heinrich J., Wichmann H.-E., Peters A. (2007). Daily mortality and particulate matter in different size classes in Erfurt, Germany. J. Expo. Sci. Environ. Epidemiol..

[20] Lanzinger S., Schneider A., Breitner S., Stafoggia M., Erzen I., Dostal M., Pastorkova A., Bastian S., Cyrys J., Zscheppang A. (2016). Associations between ultrafine and fine particles and mortality in five central European cities—Results from the UFIREG study. Environ. Int..

[21] Wolf K., Schneider A., Breitner S., Meisinger C., Heier M., Cyrys J., Kuch B., von Scheidt W., Peters A. (2015). Associations between short-term exposure to particulate matter and ultrafine particles and myocardial infarction in Augsburg, Germany. Int. J. Hyg. Environ. Health.

[22] Kettunen J., Lanki T., Tiittanen P., Aalto P.P., Koskentalo T., Kulmala M., Salomaa V., Pekkanen J. (2007). Associations of fine and ultrafine particulate air pollution with stroke mortality in an area of low air pollution levels. Stroke.

[23] Peters A., Breitner S., Cyrys J., Stölzel M., Pitz M., Wölke G., Heinrich J., Kreyling W., Küchenhoff H., Wichmann H.E. (2009). The Influence of Improved Air Quality on Mortality Risks in Erfurt, Germany.

[24] Breitner S., Su C., Franck U., Wiedensohler A., Cyrys J., Pan X., Wichmann H.E., Schneider A., Peters A. (2021). The association between particulate air pollution and respiratory mortality in Beijing before, during, and after the 2008 Olympic and Paralympic Games. Front. Environ. Sci..

[25] Bouma F., Janssen N.A.H., Wesseling J., van Ratingen S., Strak M., Kerckhoffs J., Gehring U., Hendricx W., de Hoogh K., Vermeulen R. (2023). Long-term exposure to ultrafine particles and natural and cause-specific mortality in the Netherlands. Environ. Int..

[26] Qi Q., Yu F., Nair A.A., Lau S.S.S., Luo G., Mithu I., Zhang W., Li S., Lin S. (2024). Hidden danger: The long-term effect of ultrafine particles on mortality and its sociodemographic disparities in New York State. J. Hazard. Mater..

[27] Weichenthal S., Lloyd M., Ganji A., Simon L., Xu J., Venuta A., Schmidt A., Apte J., Chen H., Lavigne E. (2024). Long-term exposure to outdoor ultrafine particles and black carbon and effects on mortality in Montreal and Toronto, Canada. Res. Rep. Health Eff. Inst..

[28] Lloyd M., Olaniyan T., Ganji A., Xu J., Venuta A., Simon L., Zhang M., Saeedi M., Yamanouchi S., Wang A. (2024). Airborne nanoparticle concentrations are associated with increased mortality risk in Canada’s two largest cities. Am. J. Respir. Crit. Care Med..

[29] Pond Z.A., Saha P.K., Coleman C.J., Presto A.A., Robinson A.L., Pope C.A. (2022). Mortality risk and long-term exposure to ultrafine particles and primary fine particle components in a national U.S. cohort. Environ. Int..

[30] Vachon J., Smargiassi A., Van Ryswyk K., Lavigne É., Anassour Laouan Sidi E., Blais C., Buteau S. (2025). Associations between all-cause and ischemic heart disease mortality and long-term ambient ultrafine particles exposure: A comparison of statistical and machine learning exposure models. Environ. Int..

[31] Simon M.C., Naumova E.N., Levy J.I., Brugge D., Durant J.L. (2020). An Ultrafine Particle Number Concentration Model for Estimating Retrospective and Prospective Long-Term Ambient Exposures in Urban Neighborhoods. Environ. Sci. Technol..

[32] He C., Morawska L., Hitchins J., Gilbert D. (2004). Contribution from indoor sources to particle number and mass concentrations in residential houses. Atmos. Environ..

[33] Lane K.J., Scammell M.K., Levy J.I., Fuller C.H., Parambi R., Zamore W., Mwamburi M., Brugge D. (2013). Positional error and time-activity patterns in near-highway proximity studies: An exposure misclassification analysis. Environ. Health.

